# Ethnic, gender and other sociodemographic biases in genome-wide association studies for the most burdensome non-communicable diseases: 2005–2022

**DOI:** 10.1093/hmg/ddac245

**Published:** 2022-10-03

**Authors:** Hugo Fitipaldi, Paul W Franks

**Affiliations:** Department of Clinical Sciences, Genetic and Molecular Epidemiology Unit, Lund University Diabetes Center, Lund University, 21428 Malmo, Sweden; Department of Clinical Sciences, Genetic and Molecular Epidemiology Unit, Lund University Diabetes Center, Lund University, 21428 Malmo, Sweden; Harvard T.H. Chan School of Public Health, Boston, MA 02115, USA

## Abstract

Introduction: Since 2005, disease-related human genetic diversity has been intensively characterized using genome-wide association studies (GWAS). Understanding how and by whom this work was performed may yield valuable insights into the generalizability of GWAS discoveries to global populations and how high-impact genetics research can be equitably sustained in the future. Materials and Methods: We mined the NHGRI-EBI GWAS Catalog (2005–2022) for the most burdensome non-communicable causes of death worldwide. We then compared (i) the geographic, ethnic and socioeconomic characteristics of study populations; (ii) the geographic and socioeconomic characteristics of the regions within which researchers were located and (iii) the extent to which male and female investigators undertook and led the research. Results: The research institutions leading the work are often US-based (37%), while the origin of samples is more diverse, with the Nordic countries having contributed as much data to GWAS as the United States (~17% of data). The majority of *first* (60%), *senior* (75%) and all (66%) authors are male; although proportions vary by disease and leadership level, male co-authors are the ubiquitous majority. The vast majority (91%) of complex trait GWAS has been performed in European ancestry populations, with cohorts and scientists predominantly located in medium-to-high socioeconomically ranked countries; apart from East Asians (~5%), other ethnicities rarely feature in published GWAS. See: https://hugofitipaldi.shinyapps.io/gwas_results/ to browse all results. Conclusion: Most GWAS cohorts are of European ancestry residing outside the United States, with a smaller yet meaningful proportion of East Asian ancestry. Papers describing GWAS research are predominantly authored by male scientists based in medium-to-high income countries.

## Graphical abstract

**Figure:**
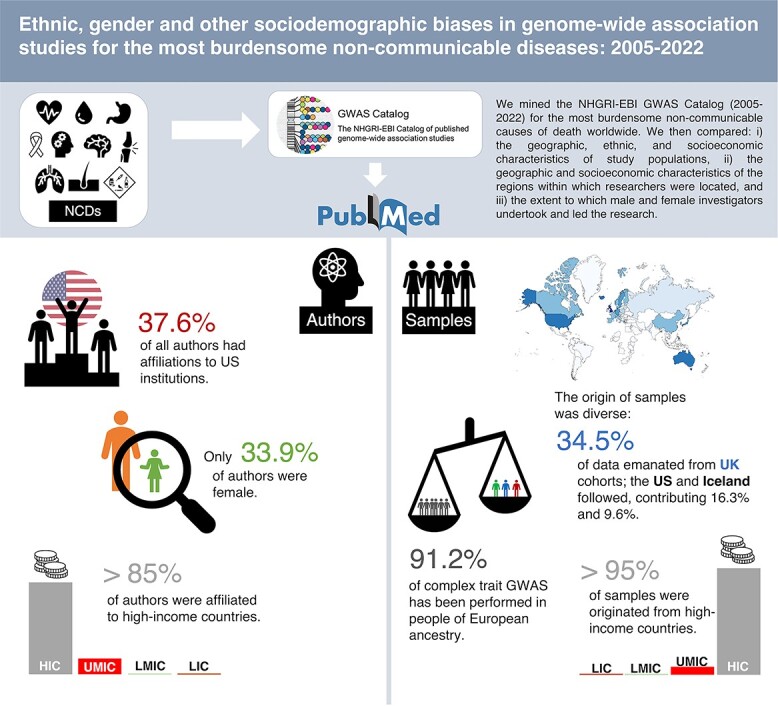

## Introduction

In the two decades since the completion of the Human Genome Project (1), human genome sequencing has provided a framework for the design of massively parallel, chip-based genotyping arrays. These technologies have been used to characterize common genomic variation in millions of people worldwide, which in turn has been used to identify tens of thousands of variants associated with complex disease through genome-wide association studies (GWAS) (2–4).

As of mid-2022, almost 6000 GWAS papers had been published (5), some of which facilitated functional studies revealing novel aspects of disease biology and highlighting therapeutic targets (6). GWAS discoveries are also of clinical value by informing the design of prediction and diagnostic tools (2,6–9) and providing markers of drug contraindication (10–14).

GWAS has predominantly focused on non-communicable diseases (NCDs) (5,15,16). In 2016, cardiovascular diseases, cancers, chronic respiratory diseases, diabetes and other NCDs accounted for 71% of deaths worldwide (17). Although NCDs are often heavily determined by social and lifestyle factors, each has a sizable genetic component (18–20).

The way GWAS have been performed, the populations within which the studies were done, and the characteristics of the scientists and institutions performing this work may impact the generalizability and translatability of GWAS discoveries to global populations; obtaining these insights may also guide strategies that help ensure the sustainability of equitable high-impact genetics research. Recent commentaries and analyses suggest that such biases indeed exist (21,22), although their specific nature and how they distribute across major disease areas have not previously been quantified.

The current analysis sought to address these issues by mining all available published GWAS papers through 2022 for the world’s most burdensome NCDs.

## Results

Out of the 5848 unique research papers in GWAS Catalog through June 2022, we identified 2300 papers where at least one of the 10 defined NCDs was listed as a disease outcome.

A Shiny web application (app) with all the results of this study is available at https://hugofitipaldi.shinyapps.io/gwas_results/.

Affiliations

**Figure 1:**
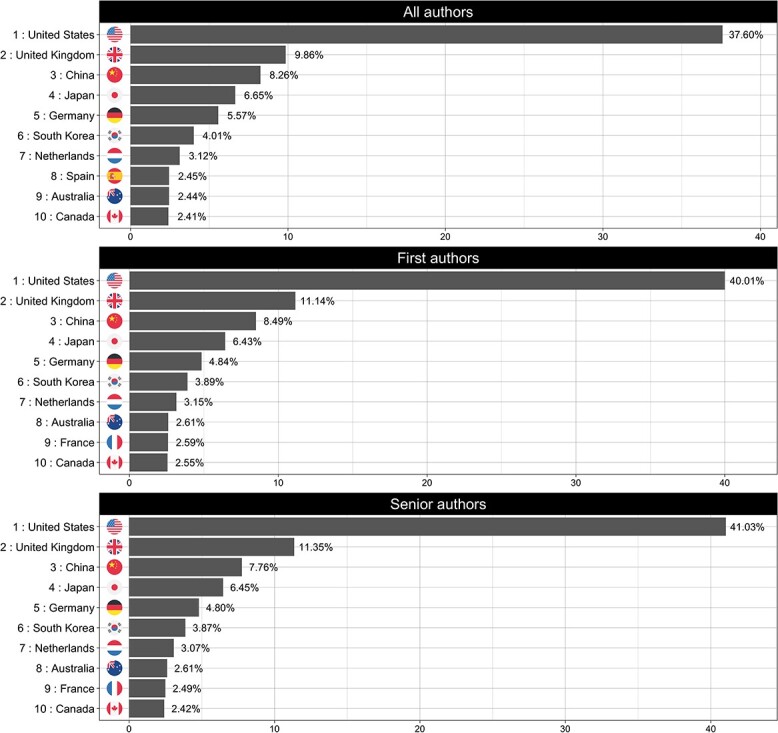
Top 10 countries of affiliation for the *All traits* disease category. The panels show the ranked list of countries of affiliation for each of the defined authors categories (*all*, *first* and *senior*).

In the analysis of the 2300 research papers from all disease areas, the United States (US) ranked top in terms of co-authorship, with more than one-third of authors (37%) having affiliations with US institutions, as shown in Figure 1. The next highest-ranking countries were the United Kingdom (UK) (9.8%), China (8.2%), Japan (6.6%) and Germany (5.5%). A very similar pattern was also observed in *first* and *senior* authorships, with the same ranking of nations, albeit with slight variations in proportions. Across all disease areas, US-affiliated *first* co-authors dominated, with 40% share of these positions (*all traits*). By clustering countries of affiliation of all authors for *all traits* in macro-regions, notably driven by the US affiliations, 40% of GWAS authors were affiliated with institutions located in North America. The next highest-ranking regions were Eastern Asia (19%) and Northern Europe (17%). Authors affiliated with institutions based in African or Latin American regions accounted for less than 1% each.

**Figure 2:**
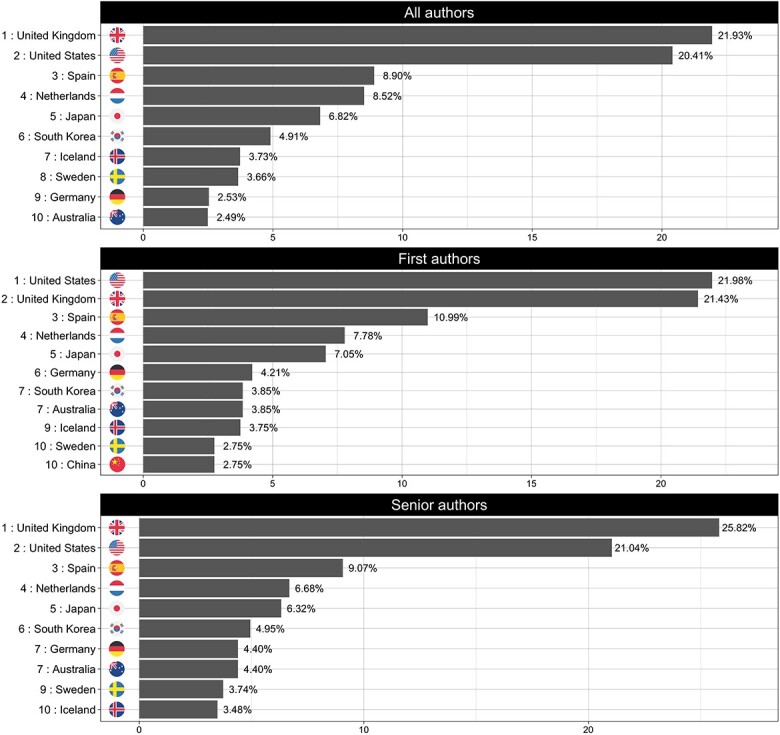
Top 10 countries of affiliation for musculoskeletal disorders disease category. The panels show the ranked list of countries of affiliation for each of the defined authors categories (*all*, *first* and *senior*).

Across almost all disease areas when examined separately, co-authors from US-based institutions dominated with a single exception: papers focusing on musculoskeletal disorders (Fig. 2), where authors affiliated with UK-based institutions are the most highly represented author group (~22%) with US-affiliated authors ranking a close second (20%).

**Figure 3:**
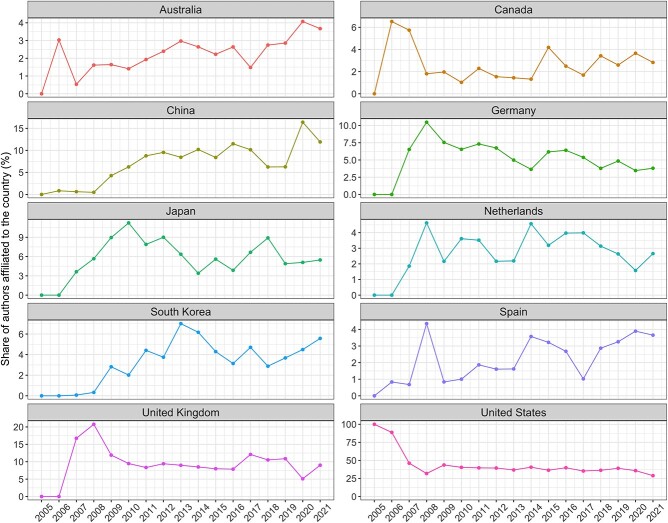
Observed trends for the top 10 countries of affiliation (*all traits* and *all authors* categories) during the analysis period (2005–2021).

Figure 3 shows the observed trends for the top 10 countries of affiliation during the analysis period. Despite ranking first over time, the US has experienced a 2.4% mean decline in authorship dominance each year (95% CI: −4.05, −0.84%; *P* < 0.01). In contrast, Australia, China, South Korea and Spain have each increased their dominance during the same period, with annual average increases of 0.14% (95% CI, 0.06, 0.22; *P* < 0.01), 0.74% (95% CI, 0.45, 1.04%; *P* < 0.01), 0.30% (95% CI, 0.13, 0.47%; *P* < 0.01) and 0.16% (95% CI, 0.05, 0.28%; *P* = 0.01), respectively. Within the top 10 countries of affiliation, no other country had a statistically significant change over time.

For all traits, 7 out of 10 top-ranking institutions according to the *ubiquity score* were from the US (Supplementary Material, Table S3a), with Harvard Medical School ranked first having fielded co-authors on 15% of GWAS publications. The second and third places are occupied by Karolinska Institutet (Sweden) and the Broad Institute (US), with 12 and 11%, respectively. For the *ubiquity score* for the *all authors* category, US-based institutions dominated the top position for most of the traits, with only three exceptions (musculoskeletal disorders, mental disorders and skin disease). The UK’s dominance in the field of musculoskeletal disorders is clear in the *ubiquity score* rankings, where the top three institutions (King’s College London, University of Manchester and Wellcome Trust Sanger Institute) are all UK-based. For mental disorders, King’s College London (UK) ranked first with 19% of authorships, and for skin disease, Karolinska Institutet (Sweden) ranked first with 14%.

Based on *dominance* rankings, Harvard Medical School (US) ranked first across publications for all traits, with 1.2% of all authors affiliated to this institution (Supplementary Material, Table S3b). deCODE genetics (Iceland) ranked first when stratified by *first* authors (2.1% of all *first* authorships). For *senior* positions, Harvard Medical School (US) also ranked first with 1.9% of affiliated authors.

### Gender

**Figure 4:**
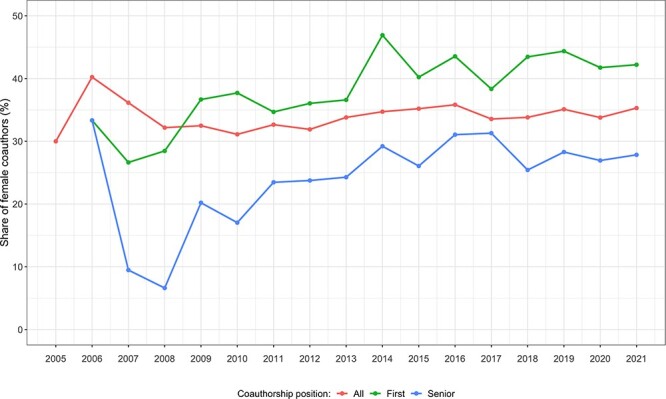
Trends of female authors in *all traits* category across the studied period (2005–2021). The figure shows changes in the representation of women in *all* (red), *first* (green) and *senior* (blue) authorship positions by year.

In the *all traits* category, 66, 60 and 75% of *all authors, first authors* and *senior authors* were males, respectively (Supplementary Material, Fig. S2). Temporal analyses revealed a consistent degree of male over-representation for all authorships during the 16-year study period, with no detectable changes in gender proportions. However, the substantial gender imbalance seen in the first 3 years of GWAS for *first* and *senior* authors has improved, with female authorship increasing significantly during the period 2005–2021 (*first authors*: χ2 = 41.45, *P* < 0.01; senior authors: χ2 = 32.6, *P* < 0.01). In 2005, all *first* and *senior authors* were male in this analysis. In 2006, females accounted for 33.33% of the *senior* position, dropping to 9.5% in 2007, and returning to 27.8% during the period 2008–2021, with an average annual increase of 0.85% (95% CI, 0.09, 1.61%; *P* = 0.03). A linear increase in females occupying *first author* positions was also observed, with an average annual increase of 0.93% (95% CI, 0.52, 1.35%; *P* < 0.01), increasing from 33.3% in 2006 to 42.2% in 2021 (Fig. 4).

Across the different disease areas, male researchers consistently occupied about two-thirds of authorships (Supplementary Material, Fig. S2). However, *first* authorships varied considerably by disease area, and while the gender of authors was most balanced for *musculoskeletal disorders* (47% female), *first* authors of *substance use* papers were predominantly male (79%). *Senior* authorships have consistently favoured male researchers, with the most balanced disease area being *musculoskeletal disorders* (65% male), and the least balanced being *chronic respiratory diseases* (79%) and *digestive diseases* (79%).

### Geographic origin of GWAS cohorts

When assessing the geographic origin of the cohorts used in published GWAS analyses, the UK ranked top with 34% of data emanating from UK cohorts; the US, Iceland, Australia and Norway followed, contributing 16, 9.6, 4.3 and 3.7 of the total data, respectively.

**Figure 5:**
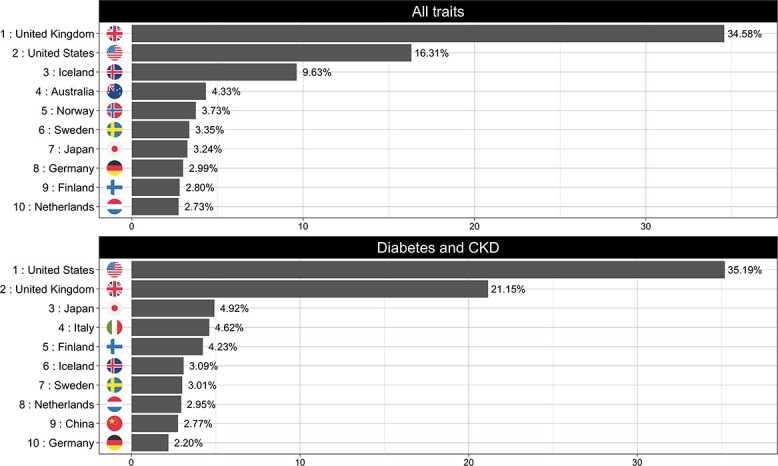
Top 10 countries of recruitment (origin of samples) for *all traits* (upper panel) and Neurological disorders (lower panel).

Although the UK ranked first when all disease areas are considered together, there was one disease-specific exception: *diabetes and chronic kidney disease* (*CKD*), in which the US ranked first with 35%, followed by the UK (21%), Japan (4.9%), Italy (4.6%) and Finland (4.2%) (Fig. 5).

**Figure 6:**
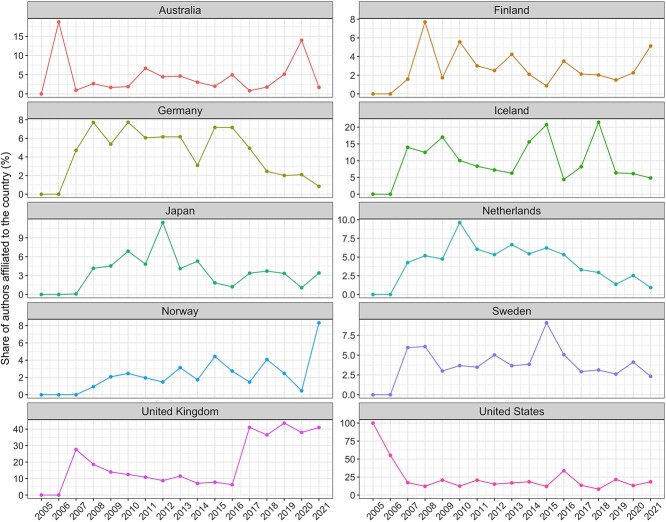
Observed trends for the top 10 countries of recruitment (*all traits*) during the analysis period (2005–2021).

Figure 6 shows the country of origin of GWAS cohorts over time. Here, Germany, Netherlands and the US contributed progressively less data year on year, declining −0.45% (95% CI, −0.65, −0.05; *P* < 0.01), −0.35% (95% CI, −0.65, −0.05; *P* = 0.02) and −0.84% (95% CI, −1.59, −0.09%; *P* = 0.02) per year, respectively. Norway and the UK on the other hand increased their data contribution by 0.14% (95% CI, 0.03, 0.25%; *P* < 0.01) and 2.13% (95% CI, 1.43, 2.84%; *P* < 0.01) per year on average. No other country’s data contributions have change significantly over time.

### Ancestry

**Figure 7:**
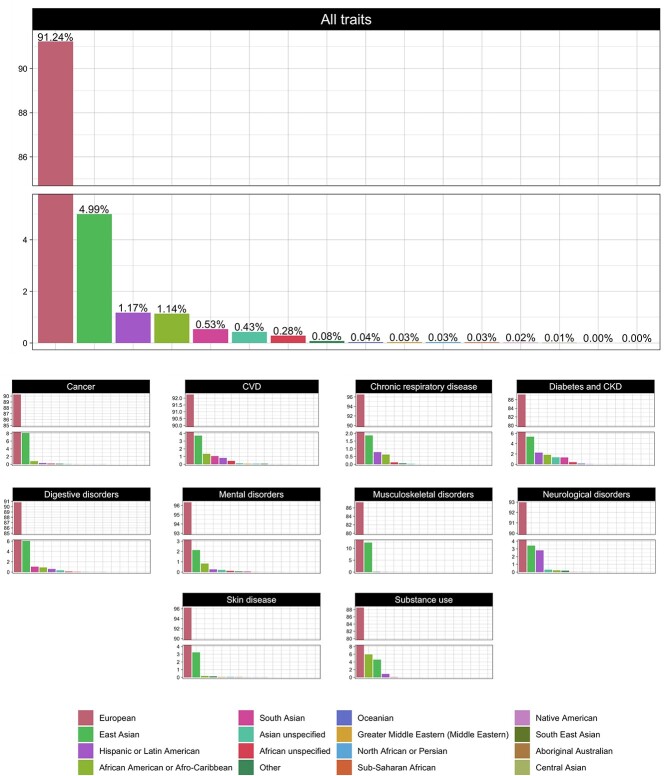
Ancestry of samples: the proportion of ancestry groups in *all traits* (upper panel) and across the 10 disease areas (lower panel).

The vast majority of GWAS analyses have been performed in people of European ancestry (91%) (Fig. 7). Overall, East Asians are the second most well represent ethnicity, with 4.9% of data. This pattern was consistent across all disease areas, with participants in most GWAS cohorts being predominantly (>90%) of European ancestry; the close exceptions are in the areas of *diabetes and CKD*, *musculoskeletal disorders* and *substance use*, were European ancestry samples comprised 87, 87 and 88% of the total samples, respectively. Although GWAS has rarely been performed in people of African ancestry (<1.5%), people of African American or Afro-Caribbean ethnicities contributed the second largest amount of data to *substance use* GWAS (5.9%). Although also dominated by European ancestry cohorts, GWAS research in the fields of *diabetes and CKD* and *digestive diseases* are the most ethnically diverse (both with 15 out of the 16 broad ancestral groups represented within their cohorts) (see Fig. 7). There are very few statistically significant temporal changes in the ethnic composition of GWAS from 2005–2021; the exceptions are ‘African unspecified’ and ‘Hispanic or Latin American’, which increased on average 2.54% per year (95% CI, 0.24, 4.84% per year; *P* = 0.03) and 8.10% (95% CI, 2.37, 13.84% per year; *P* < 0.01), respectively.

### Economic background

**Figure 8:**
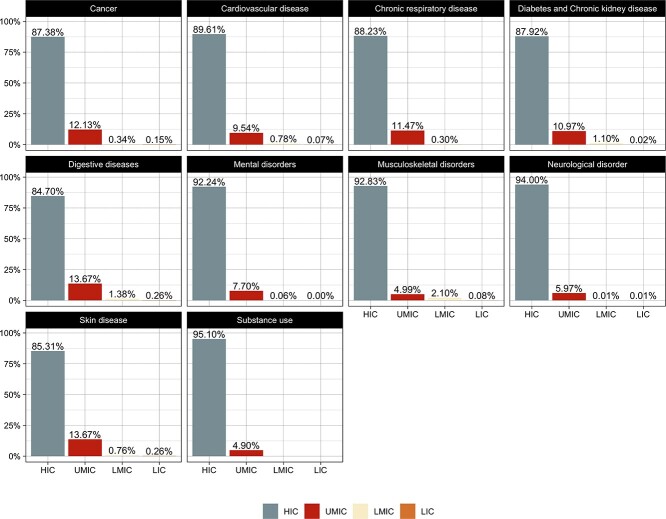
Economic background of authors. The proportion of income groups in authors (‘all authors’ position) across the 10 disease areas. Abbreviations: HIC, high-income countries; UMIC, upper-middle-income countries; LMIC, low-middle income countries; LIC, low-income countries.

Across the 10 index disease areas, the great majority of authors were classified as being affiliated to high-income countries (HIC) (Fig. 8), with the lowest percentage of this group (84%) in GWAS publications within the area of *digestive diseases*, while 95% of *substance use* GWAS research was performed by investigators based at institutions in HICs. No material differences in socioeconomic status across the different authorship categories were detected, with authors from HICs having the majority (>80%) share of authorships.

For the period 2005–2021 in the *all traits* category, the proportion of authors affiliated to HICs have been slowly declining, with average annual decreases of −0.99% per year (95% CI, −1.30, −0.68% per year; *P* < 0.01) for all authors, −0.68% per year (95% CI, −1.15, −0.21% per year; *P* < 0.01) for *first authors* and 0.90% per year (95% CI, −1.18, −0.62% per year; *P* < 0.01) for *senior authors*. Concomitantly, authors affiliated to upper-middle income countries (UMIC) have slowly increased over the years, with average annual increases of 0.91% per year (95% CI, 0.61, 1.22% per year; *P* < 0.01) for all authors, 0.64% per year (95% CI, 0.18, 1.11% per year; *P* < 0.01) for *first authors* and 0.86% per year (95% CI, 0.58, 1.14% per year; *P* < 0.01) for *senior authors*. For authors affiliated to low-middle income countries (LMIC), a statistically significant linear trend was only seen for the *all authors* category, with an average annual increase of 0.06% per year (95% CI, 0.02, 0.10% per year; *P* < 0.01). No significant trends for authors affiliated to low-income countries (LIC) were observed. Income group trends in co-authorship for specific disease areas can be explored and visualized within the results dashboard (https://hugofitipaldi.shinyapps.io/gwas_results/) results app.

**Figure 9:**
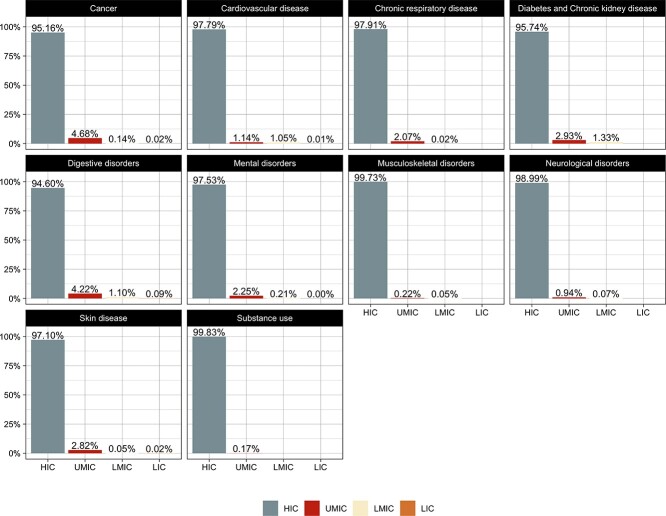
Economic background of samples. The proportion of income groups in samples across the 10 disease areas. Abbreviations: HIC, high-income countries; UMIC, upper-middle-income countries; LMIC, low-middle income countries; LIC, low-income countries.

With regard to socioeconomic ranking of study participants, HICs were overrepresented (>90%) in all disease areas (Fig. 9), with no material change during the analysis period.

## Discussion

During the past two decades, GWAS has made major contributions to understanding the biological basis of NCDs. Almost 2500 published GWAS papers address this topic to date. We extracted data from these publications using text mining and natural language processing to describe the characteristics of the cohorts that have been analyzed and the scientists who undertook this work.

The contributions of different countries, in terms of both data and authorship, vary greatly, with some countries such as Iceland having made enormous data contributions given their small populations; conversely, other large counties such as the US have dominated the leadership of published GWAS analyses, even though their *per capita* data contributions are less. We found that >90% of GWAS data come from people of European ancestry (often from the Nordic countries or the UK), with about 5% of GWAS analyses performed in people of East Asian ancestry. With few exceptions (e.g. African American and Afro-Caribbean cohorts for GWAS of *substance use*), other ethnicities have rarely featured in GWAS of NCDs. This is important because it may limit the generalizability of existing genetic discoveries to global populations (23,24). In complex disease, polygenic risk scores (PRS) have good predictive power in some clinical settings, especially for some cancers and type 1 diabetes (25,26). However, these tools sometimes translate poorly to populations of non-European ancestry (22). For example, a recent analysis from the eMERGE study (23) evaluated the performance of seven breast cancer PRSs in three distinct ancestral groups (European, African and Latinx). The authors found that these scores derived in women of European ancestry generalized well to women of Latin American ancestry, but not to African ancestry cohorts.

Nearly 90% of GWAS researchers emanated from institutions based in HICs, which is a much higher proportion than estimated by UNESCO across all research fields in 2019 (27). The US has played a leading role publishing research across many areas of health sciences over recent decades, with ~30% of publications worldwide emanating from the US in 2019 (27). Here, we found that researchers based at US institutions occupied the largest proportion of co-authorships across GWAS papers for most disease areas (~40% of authorships on average).

Reflecting the dominance of US institutions, many of the top-ranking institutions are based in the US, of which Harvard Medical School ranks first in terms of *ubiquity* regardless of authorship seniority in the analysis of all traits. Although Harvard Medical School ranks first in terms of author *dominance* for *all* and *senior* authors, it ranked second for *first authors* in the *all traits* analysis. In addition to Harvard Medical School, Harvard T.H. Chan School of Public Health, NCI and Mayo Clinic all rank within the top 10 global institutions based on the *ubiquity* score. Iceland is one of the smallest countries active in GWAS research (population ~330 000), yet a single Icelandic institution (deCODE Inc) was the second most *dominant* institution for *all* authors and had the most *first* authorships. While deCODE ranks outside the top 10 institutions in the *ubiquity score* rankings for *all* authors and *all traits*, it is ranked fourth for *first* authorships and sixth for *senior* authorships.

Although US institutions are highest ranking in terms of *ubiquity*, there are some noteworthy exceptions: The Wellcome Trust Centre for Human Genetics in Oxford (UK) ranked first for *senior* authorship of *diabetes and CKD* GWAS papers, first for *first* and *senior* authorship of *digestive diseases* GWAS papers, and *first* for *senior* authorship for GWAS of *musculoskeletal disorders*. King’s College London (UK) ranked first in the category of *all* authorship of *musculoskeletal disorders* and *mental disorders* as well as for *first* and *senior* authorships of *skin disease* GWAS papers. Karolinska Institutet (Sweden) ranked first in the *all authors* position for *skin disease*.

The prolificacy of the US likely reflects its greater capacity than other countries to generate and analyze genetic data from within and outside its borders (28), fuelled by established international collaborative networks and well-developed national funding programmes, predominantly through the National Institutes of Health. It is likely that the US will continue to dominant, as publishing success influences the extent to which national funding agencies support relevant research infrastructures (28).

Although one might imagine that authorship of papers would correspond with origin of the data, this is rarely the case. For example, more than half of the participants in *chronic respiratory disease* GWAS research hailed from the UK, with 11% from the US; in contrast, US-based scientists accounted for almost half of the *first* and *senior* authorships and UK-based scientists occupied <7% of these co-author positions. Elsewhere, in the field of *musculoskeletal disorders*, almost a quarter of GWAS participants were from Iceland, while only ~4% of *first* and *senior* authors belong to Icelandic institutions. For GWAS of *neurological disorders*, nearly 40% of the samples originated from the UK and ~20% from Australia, while only ~10% of UK-based scientists and ~2.5% of Australian-based scientists were *first* or *senior* authors on the respective papers.

Despite the success of US-based scientists in GWAS research, there has been a small, yet progressive decline in US dominance over the years. Conversely, China, South Korea and a handful of other nations are gradually increasing their leadership of GWAS research.

We observed widespread gender imbalances in co-authorship of GWAS papers, with males occupying on average about 60 and 75% of *first* and *senior* authorships, respectively. In some cases, such as *chronic respiratory disease* research, the gender ratio approached equality (56% male), yet in others like *substance use* 80% of lead *first authors* were male. Male dominance is more striking for *senior* authorships; in the most balanced area (*musculoskeletal disorders*), about one-third of lead *senior authors* are female, but only one in five *senior authors* were female in *digestive diseases* research. Nevertheless, these gender gaps progressively narrowed during the observation period and correspond with the proportion of females (roughly one-third) engaged in research *per se* during this period worldwide (27).

The current work goes considerably further than the only other publication to date on a related topic (29). It does so by providing a much deeper analysis of scientists’ affiliations, including *dominance* and *ubiquity* rankings of all participating academic institutions. We also report temporal trends for (i) author gender, (ii) author academic affiliation and (iii) ethnicity of participants. Furthermore, we used a more robust gender ascertainment method and report economic profiles of the countries where cohorts and authors reside. Moreover, all analyses were performed stratified by disease area (as well as combined), and a web-based dashboard through which all results can be accessed accompanies this paper (https://hugofitipaldi.shinyapps.io/gwas_results/).

A limitation of this analysis is that by using GWAS Catalog as our primary source of data only publications written in English were analyzed. The method used to predict author gender also has limitations; specifically, although name-to-gender inference has been validated (30), it is not a perfect proxy for self-reported gender. However, metadata from GWAS publications does not record author gender and no alternative source of these data currently exists.

In the past two decades, human genetics research has provided significant insights into the molecular basis of complex disease. This has led to improvements in drug target identification (6), clinical trial design (14) and causal inference (3). The use of GWAS data for diagnostic stratification (2,9) and prediction (2,3,22,25,26) of complex disease is also showing promise. Nevertheless, our analysis reveals that GWAS research in the most burdensome NCDs has been dominated by male researchers based at US institutions, whereas >90% of genomes analyzed are from people of European ancestry, with a large proportion of Nordic origin. Expanding leadership of human genetics research to include more female researchers and engaging researchers outside HICs should be key priorities for the future. Extending the focus of human genetics research into populations of non-European ancestry should also be prioritized, as doing so may facilitate the discovery of novel variants of clinical significance and help ensure medical tools and services designed using human genetic data are safe and efficacious, regardless of a patient’s ethnicity.

## Materials and Methods

### Selection of research papers

We selected the top 10 (of 11) non-communicable causes of death described in The Lancet’s Global Burden of Disease (GBD) ranking (2019) (31). We excluded the ninth ranked NCD (‘Other NCDs’) owing to the heterogeneous nature of the causes of death included within this term and included the 11th most burdensome cause of death, mental disorders. To identify relevant published GWAS research and published GWAS papers, we used text mining and natural language processing tools (further described in the following sections) to mine and process the NHGRI-EBI GWAS Catalog (https://www.ebi.ac.uk/gwas/, March 2005 through June 2022) (16). GWAS Catalog is a comprehensive, publicly available archive of published GWAS research and provides summary data from each publication, including details about the publication, study samples (cohort size, country of recruitment/origin of samples, ethnicity), the phenotypes investigated, and precision and magnitude of the SNP effect estimates for disease associations.

Each of the index traits was mapped to one or more Experimental Factor Ontology (EFO) reference code(s), depending on The Lancet’s definition for each disease (https://www.thelancet.com/gbd/summaries). We then used these EFOs to identify the list of GWAS within each disease area (see Supplementary Material, Table S1). Additionally, to provide a general view of GWAS research for NCDs, we included a category that encompasses all identified studies (excluding duplicates), hereby termed *All traits*.

Using study identifiers and names from the compiled list of GWAS Catalog, we gathered additional metadata (authorship information) through NCBI PubMed (www.pubmed.org) (see below for detailed explanation).

### Authors’ information

All codes relevant to the following analyses are available at: https://github.com/hugofitipaldi/gwas_mining

### Authors’ affiliations

Using R software version 4.1.2 (32), we built a tool that assembles functions to extract and pre-process co-authorship lists from the target papers (https://github.com/hugofitipaldi/affiliation). The process starts by generating a list of NCBI PubMed article identifiers (PMID) that are then used to conduct searches of PubMed. Metadata from each paper is then downloaded in an Extensible Markup Language (XML) format and through a series of text mining steps the tool retrieves the list of authors and linked affiliations.

Prior to 2014, PubMed only included the first author affiliation amongst the accessible metadata information (https://www.nlm.nih.gov/bsd/mms/medlineelements.html); thus, for papers indexed from 2005 to 2014, affiliation lists were extracted from PubMed Central (PMC) when available, or via the respective journal’s website. In these cases, a second R tool was used to link authors’ names to affiliations by creating hash dictionaries that paired the superscript digits in the co-author lists with the corresponding numbers in the lists of affiliations.

For data analysis, authors were categorized as: (i) *all authors*, comprising all authors list in each paper; (ii) *first authors* (assumed to be the more junior researchers, as per publishing convention in human genetics research), listed as the first in the author sequence or identified as joint first author with typographic marks and (iii) *senior authors*, listed as the last in the author sequence or identified as joint last author with typographic marks (assumed to be the more senior researchers, as per publishing convention in human genetics research). In the absence of typographic marks or clear statements about joint contributions for first and senior authorships, we considered authors positioned on each paper as the first and last authors as the sole *first* and *senior* authors, respectively, for that paper.

Using data mining functions written to remove noise (i.e. special characters, white spaces, URLs and punctuations) and stop-words, we reduced the original affiliation free-text field to a tidy text format (reduced corpus). To identify the institution and the country where an author was located when the work was performed, our tool utilizes *named entity recognition*, which is a natural language processing task used to identify and tag entities (organizations and locations in this case) within published texts. Institution and country of affiliation were identified by matching the identified entities with specific string terms (names of institutions, countries, states/regions, ISO codes and universities) and by geolocation using OpenStreetMap API (https://www.openstreetmap.org).

Prior to the aggregation of studies within their specific disease group, proportions of ‘country of affiliation’ were first calculated within each unique study and authors were given the same weight within their specific position (*all*, *first* or *senior* authorship). For authors with multiple affiliations, each affiliation was given equal weight, as there was no practicable solution using current text mining approaches and available databases to determine which of an author’s affiliation was primary. The final proportions of country of affiliation in each disease field were given by the aggregated sum of proportions of each country over the number of studies within each disease field (sum of all countries’ proportions).

We deployed two distinct approaches to account for the representativity of worldwide institutions in the affiliations of GWAS publications. The first, termed a *dominance score*, is derived using a similar method to the approach we used to account for country of affiliation (described above), but aggregates proportions by institutions instead of countries. The dominance score reflects the extent to which co-authors from a given institution appear on GWAS papers as a proportion of all co-authors, with institutions fielding multiple co-authors scoring better than those with few co-authors. The second approach, which we term the *ubiquity score*, expresses the frequency with which a given institutions appears within co-author affiliations across GWAS publications. In this case, institutions that have at least one co-author on many GWAS papers will score higher than those with co-authors distributed across fewer papers, regardless of the total number of co-authorships.

### Authors’ income (socioeconomic level)

To estimate the degree to which socioeconomy is related to published GWAS research, we determined the socioeconomic level of the countries within which the authors’ institutions were located using the World Bank’s calculations of gross national income per capita (in US dollars). Thereafter, the following socioeconomic categories were assigned to each author: HIC, UMIC, LMIC and LIC (https://datahelpdesk.worldbank.org/knowledgebase/articles/906519-world-bank-country-and-lending-groups).

### Authors’ gender

Each author’s gender was estimated using *genderize.io* (https://genderize.io/) and *Gender API* (https://gender-api.com/). Gender API is the most comprehensive platform available that assigns the most probable gender based on first name using more than 6 million validated names from 191 different countries. As input, queries for this API can include name (first and last) and location (country name, IP address or browser language). The API outputs gender assignments (men, women or unknown), number of samples and accuracy (from 0 to 100). The genderize.io database includes 250 000 names from 241 different countries (114 million entries in total). Queries for this service can be made with a first name and location (country) from which a probable gender is assigned where possible (men, women), count (number of samples) and probability (range 0–1). The APIs were both accessed through R software. We developed a customized R package (https://github.com/hugofitipaldi/genderAPI) through which https://gender-api.com/ can be accessed.

We parsed the co-authorship list into a series of text tidying processes that included splitting authors’ names into first and last names, removing abbreviations and accentuation. Out of the 64 061 unique author names, a small number of studies (3%) reported authorships in a format that abbreviates the authors’ first names, precluding their use in any of the tools we used to estimate gender; these papers were excluded from the gender analysis. For genderize.io, we used authors’ first names and the first country of affiliation of each author to query the API. For Gender API, we also added authors’ last names in the queries.

A comparison between name-gender classification for the 64 061 valid names that were included in both tools’ databases showed no statistical difference (see Supplementary Material, Table S2), leading us to conclude that both methods are equally valid. The APIs made equal predictions for 93% of the names. For 4% of the data one of the APIs could not make a conclusive prediction on gender (‘unknown’) and for 3% the predictions between men/women were distinct. In the first case, we used the results of the API that was able to predict gender, and for the second we choose the result that yielded higher accuracy.

### GWAS cohort participants

Information about the cohorts and participants within each GWAS paper was collected from the resources provided by GWAS Catalog (https://www.ebi.ac.uk/gwas/docs/file-downloads).

### Geographic location of the cohorts

Information about the geographic origin of the GWAS cohorts was obtained in GWAS Catalog’s ‘all ancestry data’ file. The file has an ‘origin’ field but owing to the high percentage of missing values for this variable we used the field ‘country of recruitment’ to infer sample origin. We built functions in R software to pre-process this free-text format variable, extracting countries’ names linking those to the sample size description (discovery and replication sample sizes), and excluding study populations marked as ‘Not Reported’ (10% of the data). In some papers reporting data from participants from multiple countries, insufficient information was provided to determine the country-specific contributions to the overall dataset; in these cases, we assumed that the countries have had equal shares in sample contribution.

We aggregated data by country of recruitment within each disease area and calculated the proportions of each country’s contribution, defined as the quotient between the total sample of a given country and the total sample size in the specific disease area.

### Participants’ income (socioeconomic level)

The country of origin of each cohort determined the socioeconomic category assigned to each cohort using the World Bank methodology (https://datahelpdesk.worldbank.org/knowledgebase/articles/906519-world-bank-country-and-lending-groups), as described above.

### Participants’ ancestry

We extracted ancestral information from GWAS Catalog’s *‘All ancestry data’* file (https://www.ebi.ac.uk/gwas/docs/file-downloads). GWAS Catalog formats this information in *broad* and *detailed* descriptions. Here we grouped samples by broad ancestral categories, as proposed elsewhere (33), excluding samples for which no ancestry or country of recruitment information was available (marked as ‘Not Reported’).

We pre-processed the file, passing the sample information (in free-text format) through a series of regex-based functions, to obtain information about the relative size of each broad ancestral group for each GWAS paper. Samples were aggregated around each broad ancestral category and, as with the ‘country of recruitment’ analysis, proportions were estimated within each disease area by extracting the quotient of the total sample of a given ancestry and the total sample size.

### Statistical analysis

Chi-square tests were performed to compare gender distributions within and between the three co-authorship positions. To evaluate temporal trends (per year) for gender, socioeconomy, geographic location and ethnicity, we used the Cochran-Armitage test for trend, simple linear regression, and beta regression. For the temporal trend analysis, data for 2022 are excluded because only part of the year had elapsed when analyses concluded.

## Supplementary Material

Fitipaldi_Franks_Supplementary_Information_ddac245
