# Supplementary material for: Ethnic, gender and other sociodemographic biases in genome-wide association studies for the most burdensome non-communicable diseases: 2005–2022
Source: Hum Mol Genet. 2022 Oct 3;32(3):520–32. doi: 10.1093/hmg/ddac245 (PMC9851743; doi:10.1093/hmg/ddac245)

**Supplementary material**

**Ethnic, gender and other sociodemographic biases in genome-wide association studies for the most burdensome non-communicable diseases: 2005-2022**

Hugo Fitipaldi^1^, Paul W. Franks^1,2,3*^

Affiliations

1 Department of Clinical Sciences, Genetic and Molecular Epidemiology Unit , Lund University Diabetes Center, Lund University, 21428 Malmo, Sweden

2 Harvard T.H. Chan School of Public Health, Boston, MA 02115, USA

3 Medical Science, Novo Nordisk Foundation, 2900 Copenhagen, Denmark

* To whom correspondence should be addressed at: CRC, Hus 91 plan 12, Jan Waldenströms gata 35, 214 28, Malmö, Sweden. Tel/Fax: +46 40 39 11 49; Email: paul.franks@med.lu.se

**Supplementary table 1.** Disease areas and EFO traits

| **Disease** | **EFO** | **n (GWAS Catalog)** |
| --- | --- | --- |
| **Cardiovascular disease** | EFO_0000319 | 421 |
| **Cancer** | EFO_0000311 | 545 |
| **Chronic respiratory diseases** | EFO_0000684 | 272 |
| **Diabetes and chronic kidney disease (CKD)** | EFO_0000400  EFO_0003884 | 223 |
| **Digestive diseases** | EFO_0000405 | 580 |
| **Mental disorders** | EFO_0000677 | 492 |
| **Muculoskeletal disorders** | EFO_0000685  EFO_0002506/MONDO_0005178 | 91 |
| **Neurological disorders** | EFO_0000249/MONDO_0004975  EFO_0002508/MONDO_0005180  EFO_0000474  EFO_0003885/MONDO_0005301  EFO_0003821/MONDO_0005277 | 219 |
| **Skin diseases** | EFO_0000701 | 186 |
| **Substance use** | EFO_0003829/MONDO_0007079  EFO_0003768  EFO_0003890 | 60 |

**Supplementary table 2.** Comparison of name-gender classification tools’ predictions on all authors (AA), first authors (FA) and senior authors (SA) across disease areas.

|  | **genderAPI** | |  | **genderize.io** | | |  | |  |
| --- | --- | --- | --- | --- | --- | --- | --- | --- | --- |
|  | **AA (%)** | **FA (%)** | **SA (%)** | **AA (%)** | | **FA (%)** | | **SA (%)** | **X2 (p-value)AA/ X2 (p-value)FA/ X2 (p-value)SA** |
| **All traits** |  |  |  |  |  | |  | |  |
| Female | 31,25 | 36,36 | 23,27 | 31,2 | 37,11 | | 24,42 | | 0.00(1) / 0.00(1) / 0.00(0.98) |
| Male | 61,24 | 55,83 | 69,58 | 58,57 | 52,32 | | 65,77 | |  |
| unknown | 7,51 | 7,81 | 7,14 | 10,23 | 10,57 | | 9,81 | |  |
| **Cancer** |  |  |  |  |  | |  | |  |
| Female | 35,49 | 41,63 | 26,24 | 35,85 | 43,26 | | 30,57 | | 0.00(1) / 0.00(0.92) / 0.27(0.60) |
| Male | 60,76 | 54,54 | 70,53 | 58,12 | 51,89 | | 64,26 | |  |
| unknown | 3,75 | 3,83 | 3,23 | 6,04 | 4,85 | | 5,17 | |  |
| **Cardiovascular disease** | |  |  |  |  | |  | |  |
| Female | 30,68 | 36,69 | 26,02 | 30,91 | 37,78 | | 26,21 | | 0.00(1) / 0.00(0.98) / 0.00(1) |
| Male | 65,8 | 59,65 | 70,89 | 62,09 | 54,18 | | 66,25 | |  |
| unknown | 3,52 | 3,66 | 3,08 | 7,01 | 8,04 | | 7,54 | |  |
| **Chronic respiratory disease** | | |  |  |  | |  | |  |
| Female | 30,35 | 38,53 | 18,92 | 29,76 | 41,55 | | 20,3 | | 0.00(1) / 0.08(0.77) / 0.00(0.94) |
| Male | 61,92 | 53,51 | 74,46 | 59,29 | 47,38 | | 70,48 | |  |
| unknown | 7,73 | 7,97 | 6,62 | 10,95 | 11,07 | | 9,22 | |  |
| **Diabetes and CKD** | |  |  |  |  | |  | |  |
| Female | 32,65 | 36,84 | 22,38 | 32,68 | 38,05 | | 21,8 | | 0.00(1) / 0.00(0.97) / 0.00(1) |
| Male | 61,97 | 57,88 | 72,69 | 58,32 | 53,75 | | 68,05 | |  |
| unknown | 5,38 | 5,28 | 4,92 | 9 | 8,2 | | 10,15 | |  |
| **Digestive diseases** | |  |  |  |  | |  | |  |
| Female | 29,2 | 33,4 | 20,13 | 29,46 | 34,64 | | 19,56 | | 0.00(1) / 0.00(0.97) / 0.00(1) |
| Male | 65,45 | 61,73 | 74,59 | 61,75 | 57,49 | | 71,17 | |  |
| unknown | 5,35 | 4,87 | 5,28 | 8,8 | 7,86 | | 9,27 | |  |
| **Mental disorders** |  |  |  |  |  | |  | |  |
| Female | 29,25 | 30,79 | 22,73 | 28,67 | 30,22 | | 21,95 | | 0.00(1) / 0.00(1) / 0.00(1) |
| Male | 55,69 | 52,72 | 62,7 | 54,21 | 50,74 | | 62,39 | |  |
| unknown | 15,05 | 16,5 | 14,57 | 17,13 | 19,04 | | 15,66 | |  |
| **Musculoskeletal disorders** | |  |  |  |  | |  | |  |
| Female | 34,23 | 44,96 | 32,47 | 33,78 | 43,86 | | 34,49 | | 0.00(1) / 0.00(0.98) / 0.02(0.87) |
| Male | 59,1 | 47,89 | 61,48 | 58,9 | 45,7 | | 58 | |  |
| unknown | 6,68 | 7,14 | 6,04 | 7,32 | 10,44 | | 7,51 | |  |
| **Neurological disorders** | |  |  |  |  | |  | |  |
| Female | 32,23 | 33,49 | 21,53 | 31,73 | 31,85 | | 21,46 | | 0.00(1) / 0.00(0.92) / 0.00(1) |
| Male | 58,29 | 54,38 | 69,64 | 57,46 | 54,83 | | 66,99 | |  |
| unknown | 9,48 | 12,14 | 8,83 | 10,81 | 13,32 | | 11,55 | |  |
| **Skin disease** |  |  |  |  |  | |  | |  |
| Female | 33,21 | 36,21 | 24,41 | 32,85 | 37,77 | | 29,14 | | 0.00(1) / 0.01(0.93) / 0.35(0.55) |
| Male | 61,49 | 58,84 | 70,22 | 57,86 | 53,07 | | 62,45 | |  |
| unknown | 5,3 | 4,95 | 5,38 | 9,29 | 9,16 | | 8,41 | |  |
| **Substance abuse** | |  |  |  |  | |  | |  |
| Female | 24,03 | 18,61 | 25,83 | 23,15 | 16,94 | | 27,5 | | 0.00(1) / 0(0.90) / 0.01(0.91) |
| Male | 58,76 | 55,83 | 60,83 | 55,64 | 59,72 | | 55 | |  |
| unknown | 17,21 | 25,56 | 13,33 | 21,21 | 23,33 | | 17,5 | |  |

**Supplementary table 3a.** Top-20 institutions of affiliation all traits (all authors) - ubiquitous score

| **Rank** | **Institution** | **ubiquitous score (%)** |
| --- | --- | --- |
| **1** | Harvard Medical School, Boston, United States | 15,5 |
| **2** | Karolinska Institutet, Stockholm, Sweden | 12,5 |
| **3** | Broad Institute, Cambridge, United States | 10,9 |
| **4** | Johns Hopkins University, Baltimore, United States | 10,7 |
| **5** | University of Cambridge, Cambridge, United Kingdom | 10,4 |
| **6** | Harvard T.H. Chan School of Public Health, Boston, United States | 10,2 |
| **7** | University of Texas, Houston, United States | 10 |
| **8** | Mayo Clinic, Rochester, United States | 9,6 |
| **8** | University of Washington, Seattle, United States | 9,6 |
| **10** | Inserm, Paris, France | 9,5 |
| **10** | University of Pennsylvania, Philadelphia, United States | 9,5 |
| **12** | King's College London, London, United Kingdom | 9,2 |
| **13** | National Cancer Institute (NCI), Bethesda, United States | 9 |
| **14** | University of Southern California, Los Angeles, United States | 8,6 |
| **15** | University of California, San Francisco, San Francisco, United States | 8,4 |
| **15** | Erasmus MC, Rotterdam, Netherlands | 8,4 |
| **17** | University of Oxford, Oxford, United Kingdom | 8,3 |
| **18** | University of Toronto, Toronto, Canada | 8,2 |
| **18** | The University of North Carolina at Chapel Hill, Chapel Hill, United States | 8,2 |
| **20** | Vanderbilt University School of Medicine, Nashville, United States | 8 |

**Supplementary table 3b.** Top-20 institutions of affiliation all traits (all authors) - dominance score

| **Rank** | **Institution** | **dominance score (%)** |
| --- | --- | --- |
| **1** | Harvard Medical School, Boston, United States | 1,212 |
| **2** | deCODE genetics, Reykjavik, Iceland | 1,119 |
| **3** | Karolinska Institutet, Stockholm, Sweden | 1,099 |
| **4** | Mayo Clinic, Rochester, United States | 1,061 |
| **5** | Johns Hopkins University, Baltimore, United States | 1,046 |
| **6** | National Cancer Institute (NCI), Bethesda, United States | 0,991 |
| **7** | King's College London, London, United Kingdom | 0,909 |
| **8** | Broad Institute, Cambridge, United States | 0,9 |
| **9** | Duke University, Durham, United States | 0,866 |
| **10** | University of California, San Francisco, San Francisco, United States | 0,831 |
| **11** | Harvard T.H. Chan School of Public Health, Boston, United States | 0,83 |
| **12** | University of Pennsylvania, Philadelphia, United States | 0,802 |
| **13** | Nanjing Medical University, Nanjing, China | 0,785 |
| **14** | University of Michigan, Ann Arbor, Ann Arbor, United States | 0,743 |
| **15** | The University of North Carolina at Chapel Hill, Chapel Hill, United States | 0,737 |
| **16** | Erasmus MC, Rotterdam, Netherlands | 0,726 |
| **17** | University of Cambridge, Cambridge, United Kingdom | 0,721 |
| **18** | Inserm, Paris, France | 0,717 |
| **19** | University of Toronto, Toronto, Canada | 0,681 |
| **20** | The University of Tokyo, Tokyo, Japan | 0,675 |

**Supplementary figure 1.** Flowchart of data collection and pre-processing methods.


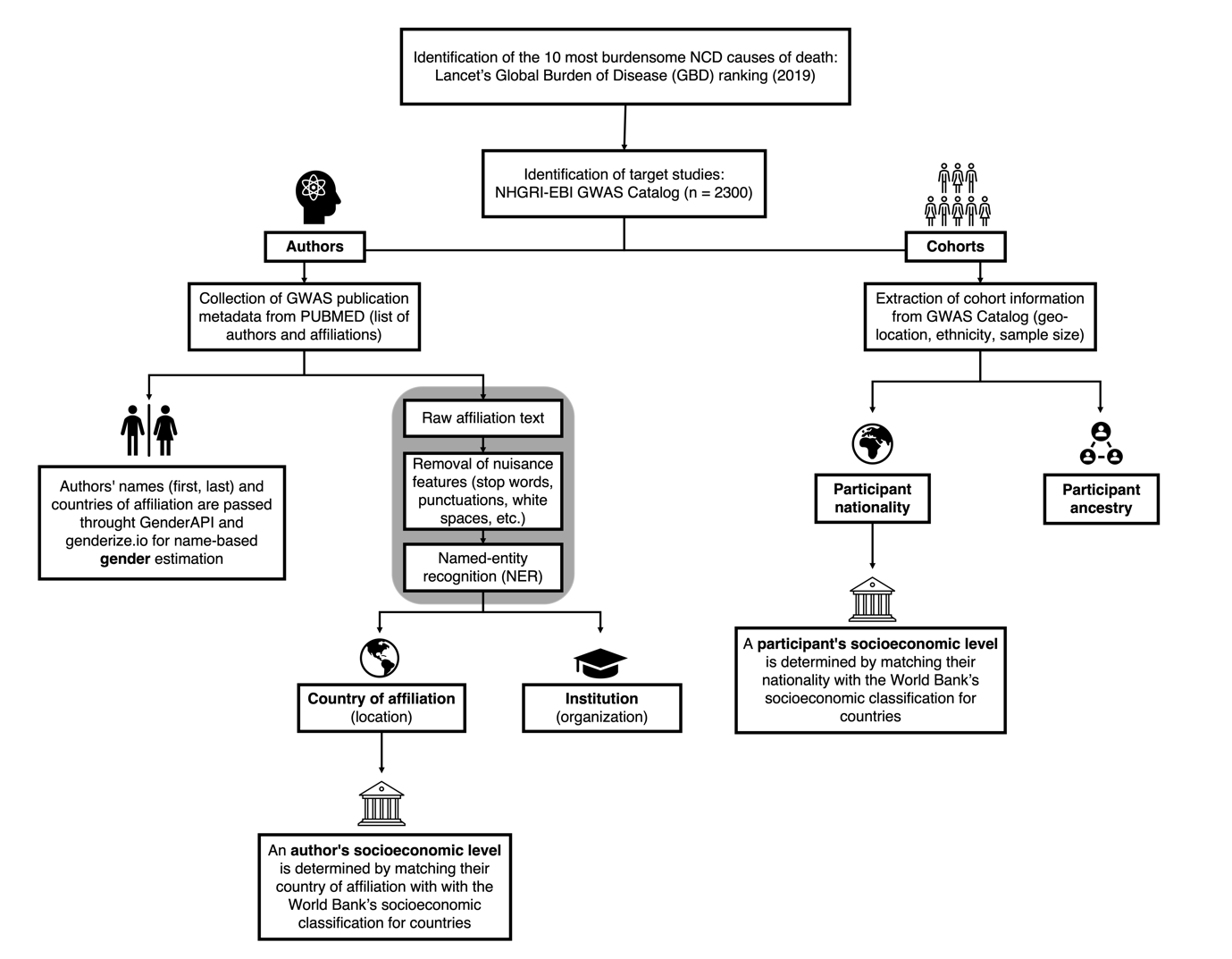


**Supplementary figure 2. Gender distributions of authors. The proportion of male (orange) and female (green) authors across disease areas and authorship positions: all (left), first (middle) and senior (right) panels.**

**
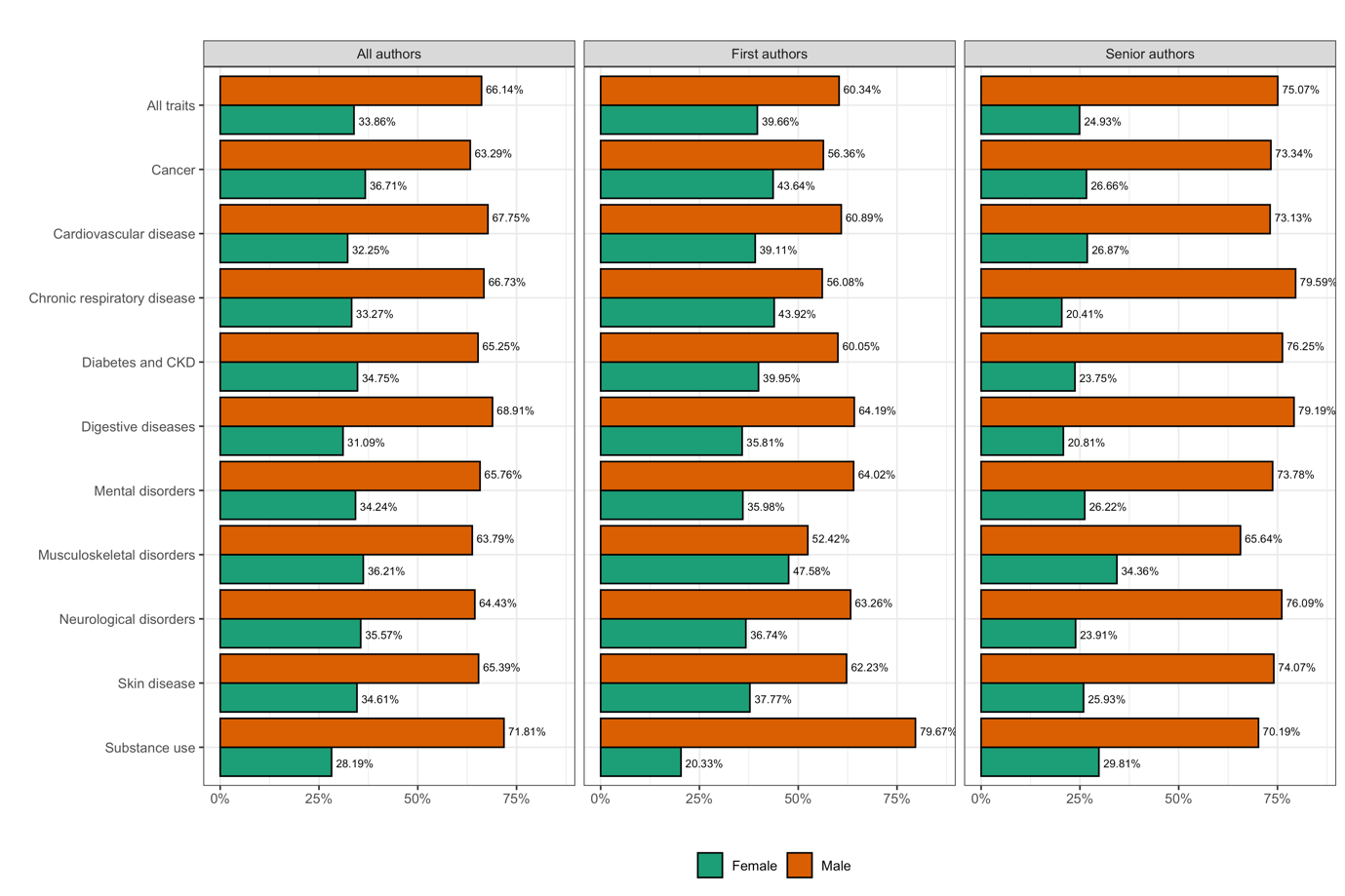
**

**Supplementary figure 3a.** Top 5 rank country of affiliations across disease areas – all authors.


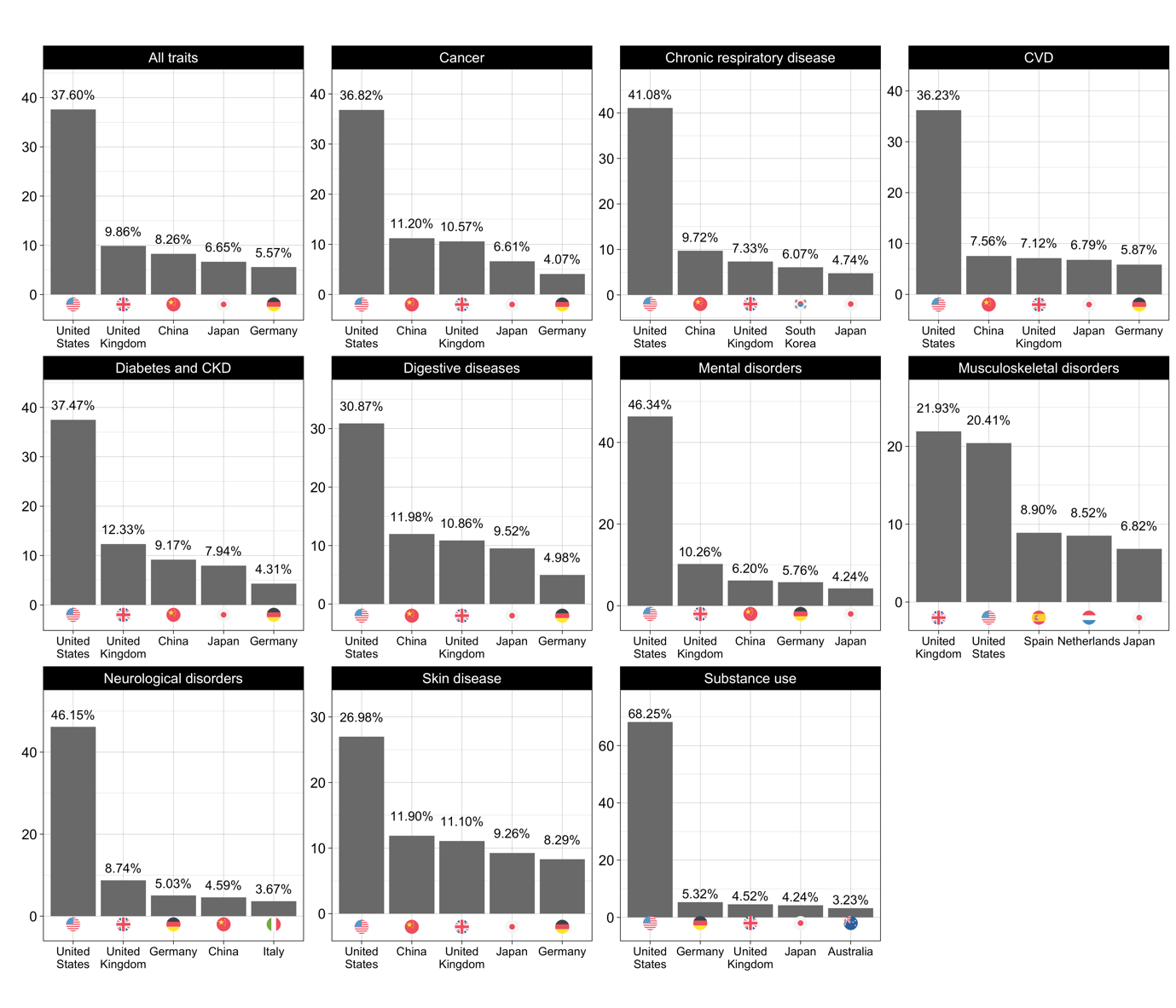


**Supplementary figure 3b.** Top 5 rank country of affiliations across disease areas – first authors.


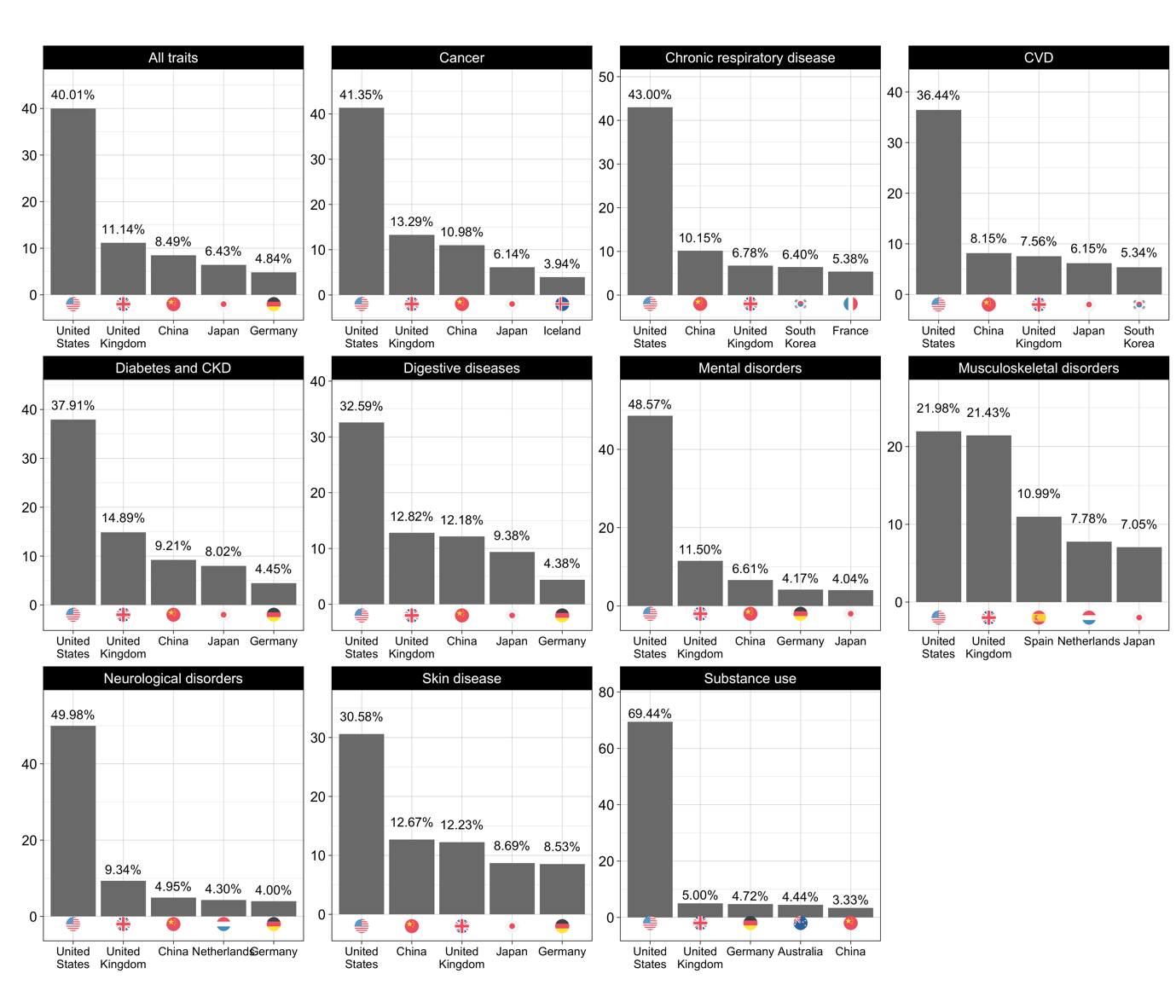


**Supplementary figure 3c.** Top 5 rank country of affiliations across disease areas – senior authors.


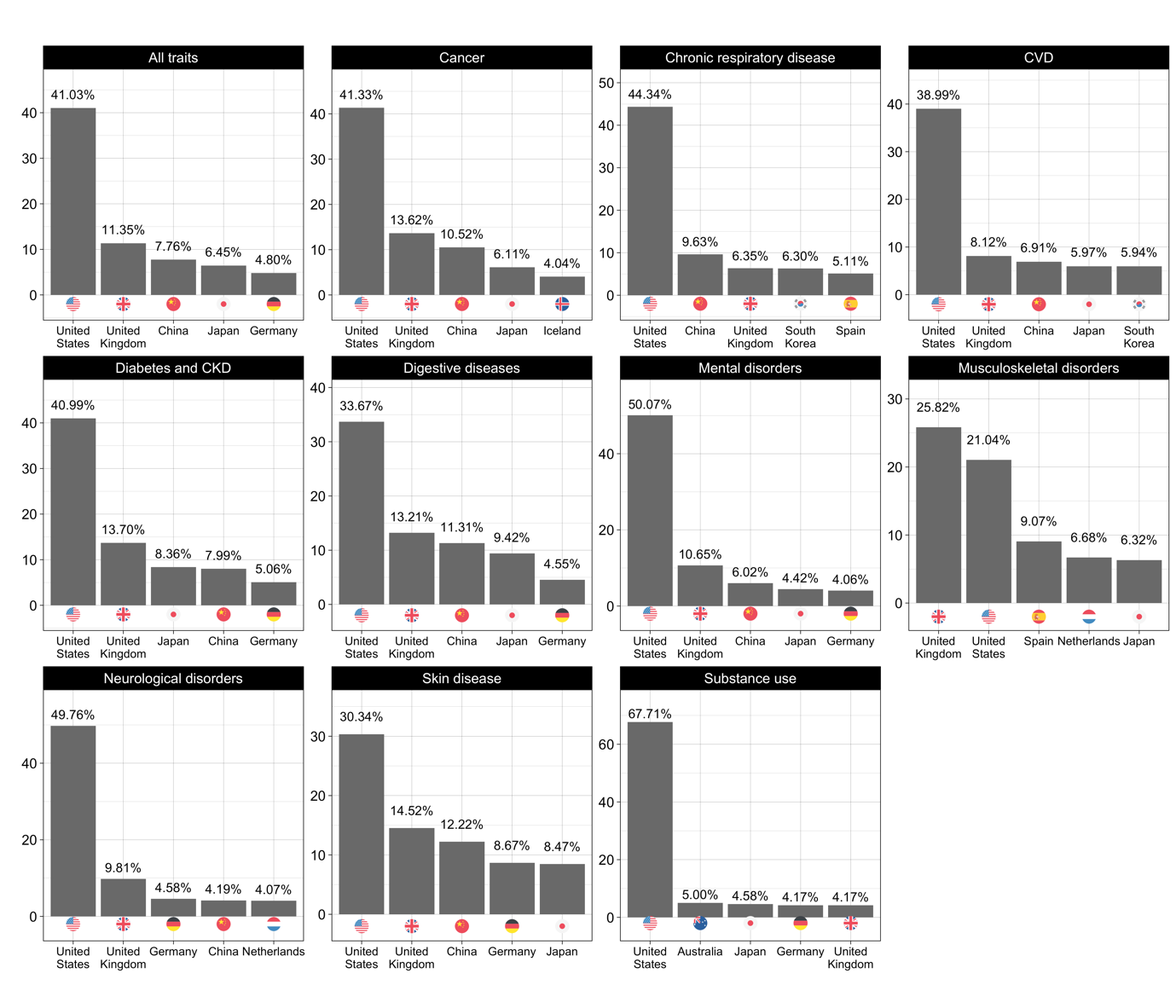


**Supplementary figure 4.** Top 5 rank country of recruitment across disease areas


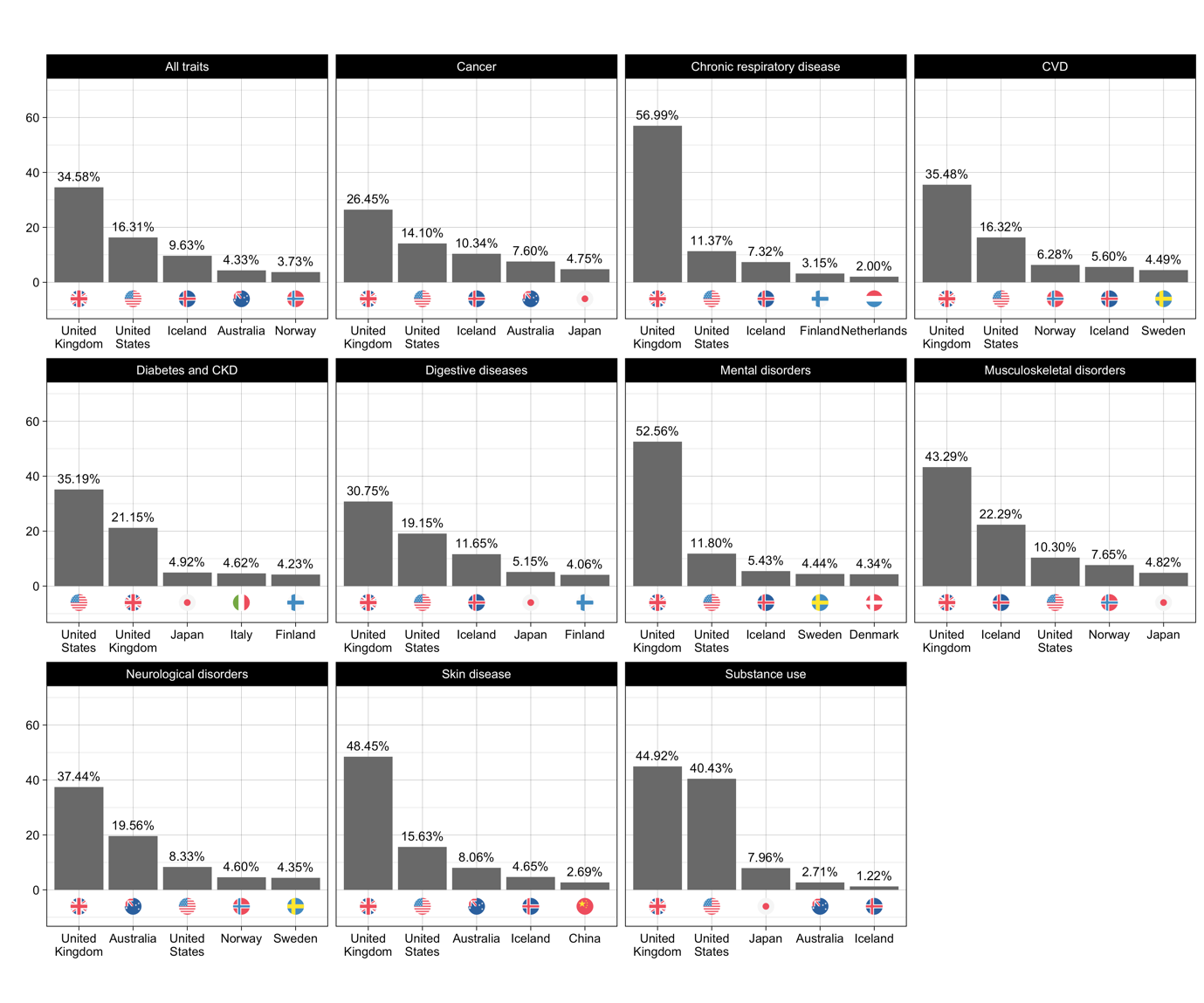

Supplement: Fitipaldi_Franks_Supplementary_Information_ddac245 [file fitipaldi_franks_supplementary_information_ddac245.doc]
